# Identification of CD8^+^ T-cell exhaustion signatures for prognosis in HBV-related hepatocellular carcinoma patients by integrated analysis of single-cell and bulk RNA-sequencing

**DOI:** 10.1186/s12885-023-11804-3

**Published:** 2024-01-10

**Authors:** Jianhao Li, Han Chen, Lang Bai, Hong Tang

**Affiliations:** 1 Center of Infectious Diseases, West China Hospital of Sichuan University, Chengdu, 610041 China; 2 Division of Infectious Diseases, State Key Laboratory of Biotherapy and Center of Infectious Diseases, West China Hospital of Sichuan University, Chengdu, 610041 China

**Keywords:** HBV-related hepatocellular carcinoma, CD8^+^ T cell exhaustion signatures, Prognosis, Immunotherapy

## Abstract

**Background:**

HBV infection is the leading risk factor for HCC. HBV infection has been confirmed to be associated with the exhaustion status of CD8^+^ T cells and immunotherapeutic efficacy in HCC. In this study, we aimed to investigate the prognostic value of the CD8^+^ T-cell exhaustion signature and immunotherapy response in patients with HBV-related HCC.

**Methods:**

We identified different clusters of HBV-related HCC cells by single-cell RNA sequencing (scRNA-seq) and identified CD8^+^ T-cell exhaustion-related genes (TERGs) by pseudotime analysis. We conducted differential expression analysis and LASSO Cox regression to detect genes and construct a CD8^+^ T-cell exhaustion index (TEI). We next combined the TEI with other clinicopathological factors to design a prognostic nomogram for HCC patients. We also analysed the difference in the TEI between the non-responder and responder groups during anti-PD-L1 therapy. In addition, we investigated how HBV induces CD8^+^ T lymphocyte exhaustion through the inhibition of tyrosine metabolism in HCC using gene set enrichment analysis and RT‒qPCR.

**Results:**

A CD8^+^ T-cell exhaustion index (TEI) was established with 5 TERGs (EEF1E1, GAGE1, CHORDC1, IKBIP and MAGOH). An AFP level > 500 ng, vascular invasion, histologic grade (G3-G4), advanced TNM stage and poor five-year prognosis were related to a higher TEI score, while HBV infection was related to a lower TEI score. Among those receiving anti-PD-L1 therapy, responders had lower TEIs than non-responders did. The TEI also serves as an independent prognostic factor for HCC, and the nomogram incorporating the TEI, TNM stage, and vascular invasion exhibited excellent predictive value for the prognosis in HCC patients. RT‒qPCR revealed that among the tyrosine metabolism-associated genes, TAT (tyrosine aminotransferase) and HGD (homogentisate 1,2 dioxygenase) were expressed at lower levels in HBV-HCC than in non-HBV HCC.

**Conclusion:**

Generally, we established a novel TEI model by comprehensively analysing the progression of CD8^+^ T-cell exhaustion, which shows promise for predicting the clinical prognosis and potential immunotherapeutic efficacy in HBV-related HCC patients.

**Supplementary Information:**

The online version contains supplementary material available at 10.1186/s12885-023-11804-3.

## Introduction

Hepatocellular carcinoma (HCC) is one of the most common cancers and is the fourth leading cause of cancer death worldwide [1]. Chronic infections with hepatitis B virus (HBV) or hepatitis C virus (HCV) are the foremost risk factors for the emergence of HCC [2]. At present, the primary treatment modalities for HCC are surgical resection and percutaneous ethanol injection [3]. Recent immunotherapies, such as immune checkpoint inhibitors, have demonstrated promising clinical outcomes in patients with HCC [4]. However, patient response rates vary, with only approximately 20% of patients achieving positive treatment outcomes [5, 6]. Despite significant efforts devoted to the treatment and management of HCC, the 5-year overall survival (OS) rate has remained low [1]. This poor prognosis has been linked to late-stage diagnosis, tumor recurrence, and inadequate treatment options [7]. Thus, there is an urgent need to develop potent prognostic predictors and novel therapeutic strategies to improve the diagnosis and treatment of HBV-related HCC.

The tumor microenvironment (TME) comprises an array of immune cells that infiltrate the liver and establish distinct immune niches, with interactions with stromal cells affecting the differentiation, tumorigenesis, and development of HCC [8–11]. Furthermore, since HCC is an inflammation-driven disease, the severity, status, and dynamic interplay of immune cells can significantly impact the prognosis and effectiveness of immunotherapy [12–14]. For example, CD8^+^ T effector cells (Teffs) play a critical role in tumor control and contribute to a better prognosis in HCC. As the main cause of HCC, HBV infection has been confirmed to be associated with the infiltration and exhaustion status of CD8^+^ T cells [15]. Continuous antigen stimulation may lead to CD8^+^ Teff cells exhaustion, resulting in impaired antitumor immunity in HBV-related HCC [16]. The advent of single-cell RNA-sequencing (scRNA-seq) has revolutionized the comprehensive profiling of the immune system, enabling a deeper understanding of immune cell heterogeneity and diversity [17]. A recent study generated a single-cell atlas of the multicellular ecosystem of HCC while also characterizing the heterogeneous subpopulations of malignant cells and their multifaceted functions in shaping the immune microenvironment of HCC [18]. Additionally, the present study evaluated the relationship between HBV infection and T-cell infiltration and exhaustion [18]. Nonetheless, the prognostic value and mechanisms of exhaustion of CD8^+^ T cells and their subsets in HBV-related HCC are unclear.

**Fig. 1:**
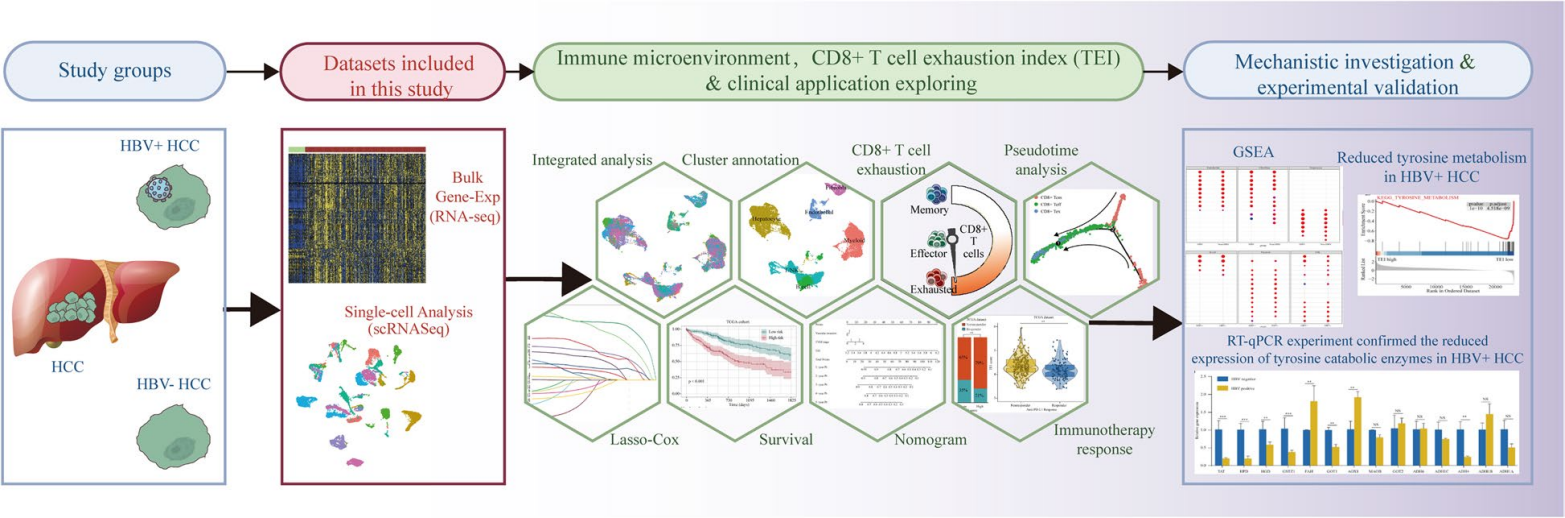
Flowchart for comprehensive analysis of CD8^+^ T cell exhaustion in HBV-related HCC

In this study, we combined scRNA-seq and Bulk RNA-Seq to investigate the ability of the CD8^+^ T-cell exhaustion signature to predict the prognosis and immunotherapy response in HBV-related HCC patients (Fig. 1). First, we annotated HCC cell types based on marker genes. Subsequently, differential expression analysis was performed to compare the proportions of cell types between HBV-positive and negative HCC samples. We observed an increase in the proportion of CD8^+^ memory T cells (Tem) and exhausted T cells (Tex), and a decrease in the proportion of Teff cells in HBV-positive HCC samples. To further explore the underlying mechanisms, we employed pseudotime analysis to identify key genes involved in the transition between different functional states of CD8^+^ T cells. Differential analysis and univariate Cox regression were also conducted on genes related to exhaustion of CD8^+^ T cells (TERGs) to detect genes that exhibited differential expression in HBV-associated HCC and were significantly linked to HCC prognosis. We established the CD8^+^ T-cell exhaustion index (TEI) by utilizing least absolute shrinkage and selection operator (LASSO) regression. We then integrated the TEI with other clinicopathological factors to design a prognostic nomogram for HCC patients. Additionally, we explored the potential mechanism underlying the promotion of CD8^+^ T-cell exhaustion due to HBV infection via the modulation of tyrosine metabolism. In summary, our study provides novel insights into the immunology of HBV-related HCC and offers valuable guidance for prognosis assessment and immunotherapy selection in the management of HCC.

## Materials and methods

### Data source and preprocessing

The HCC scRNA-seq dataset GSE149614, containing 10 primary tumor (PT) patients, was downloaded from the Gene Expression Omnibus (GEO) database (https://www.ncbi.nlm.nih.gov/geo/) [18]. From these samples, we selected five HBV-positive and two HBV-negative samples for downstream analysis. The original dataset comprised 24,859 cells and 25,479 genes. Quality control procedures were performed using the PercentageFeatureSet function to assess the percentage of mitochondrial and rRNA content in each cell. Cells expressing between 200 and 6000 genes and with less than 20% mitochondrial content were retained, while cells with a minimum of 500 Unique Molecular Identifiers (UMIs) were included in subsequent analyses.

Public clinical data and gene expression information were obtained from the TCGA (https://portal.gdc.cancer.gov/) and International Cancer Genome Consortium (ICGC) databases (https://xena.ucsc.edu/). A total of 367 samples from the TCGA-LIHC cohort and 232 samples from the ICGC-JP cohort were included for further analysis.

### Data integration, clustering and cell type identification

First, we normalize the merged data through log-normalization and find the first 2000 highly variable genes through the FindVariableFeatures function. All genes were scaled using the ScaleData function, and the RunPCA function was applied to reduce the dimensionality of PCA for the top 2000 highly variable genes selected earlier. Subsequently, we used the RunHarmony function to remove batch effects between different samples. We choose dim = 20 and clustered the cells through the “FindNeighbors” and “FindClusters” functions (resolution = 0.6) to find the cell clusters. Next, we selected the top 20 principal components to further reduce dimensionality using the UMAP (Uniform Manifold Approximation and Projection) method. Next, through the function FindAllMarkers, groups of over expressed genes were identified to find subclusters. All UMAP visualizations, violin plots, and feature plots in the paper were produced using Seurat [19] functions in conjunction with the ggplot2, and pheatmap R packages. Finally, Cellmarker2.0 was employed to extract markers for different cell types, and the resulting annotation was used as a reference for subsequent analyses.

### Trajectory analysis

To investigate the developmental trajectory of CD8^+^ T cells during tumor development and progression, we conducted pseudotime analysis using Monocle (version 3.14.0) on the gene expression matrix annotated with Seurat [20]. We identified differentially expressed genes between HBV-positive and HBV-negative HCC in CD8^+^ T cells and arranged the cells along a pseudotime trajectory. Heatmaps were generated based on genes expressed at each branch of the trajectory, and the cells were clustered into four groups based on their gene expression patterns.

### Identification and validation of TEI

Twenty TERGs were LASSO Cox regression model to construct the powerful prognostic signature. Finally, five regulators and their coefficients were selected and use to construct a prediction model. The risk score was calculated using the following formula:

$$\mathrm{Risk}\;\mathrm{score}\;=\;\sum_{\mathrm i=1}^{\mathrm n}\mathrm{Coefi}\;\ast\;\mathrm{xi}$$

where, Coefi is the coefficient and xi is the mRNA expression value of five regulators. This formula was used to calculate the TEI for every patient in both the training (TCGA) and validation (ICGC) datasets. The predictive ability of the TEI on five-year prognosis in HCC patients was evaluated by calculating the time-dependent area under the curves (AUC).

### GO and KEGG pathway functional enrichment analysis

GO annotation and KEGG pathway enrichment analyses were executed utilizing R package - clusterProfiler (version 3.14.3) on the basis of hypergeometric distribution [21–23]. The adjusted *p*-value was estimated through the Benjamini and Hochberg approach, where P.adjust value < 0.05 was recognized as statistically substantial. Display of the outcomes was achieved by invoking the dotplot utility in clusterProfiler.

### Samples collection

This study conducted experimental validation on fresh postoperative tumor tissues from three HCC patients, including three HBV-negative and HBV-positive individuals, who were admitted to West China Hospital from January to September 2023.These patients did not receive prior radiotherapy or chemotherapy and underwent HCC surgery confirmed by pathological examination.

### Quantitative polymerase chain reaction

Total RNA was extracted from these samples using TRIzol reagent (Life Technologies, CA, United States) and cDNA was generated by utilizing the SuperScript III First-Strand Synthesis System. qRT-PCR was executed on the six System (ABI, Foster City, CA, United States) employing the PowerUp SYBR Green kit (ABI, Foster City, CA, United States). The 2^-ΔΔCt method was employed to estimate relative gene expression. Supplementary Table S1 presents the primer sequences implemented in this study.

### Statistical analysis

Unless specified otherwise, all statistical analyses were executed employing R (version 4.0.4). Student’s t-test (two-tailed, unpaired) was invoked to distinguish differences between two independent groups. To scrutinize the correlation between TEI and clinical features, the Chi-square test was employed. The median TEI value was used to conduct Kaplan-Meier analyses for OS, applying the log-rank test. Univariate and multivariate Cox regression analyses were executed to identify the relationships between different variables and clinical outcome. *P*-value < 0.05 was regarded as statistically significant.

### Result

#### Identification of clusters of HBV-related HCC cells using scRNA-seq data exhibiting high cell heterogeneity

**Fig. 2:**
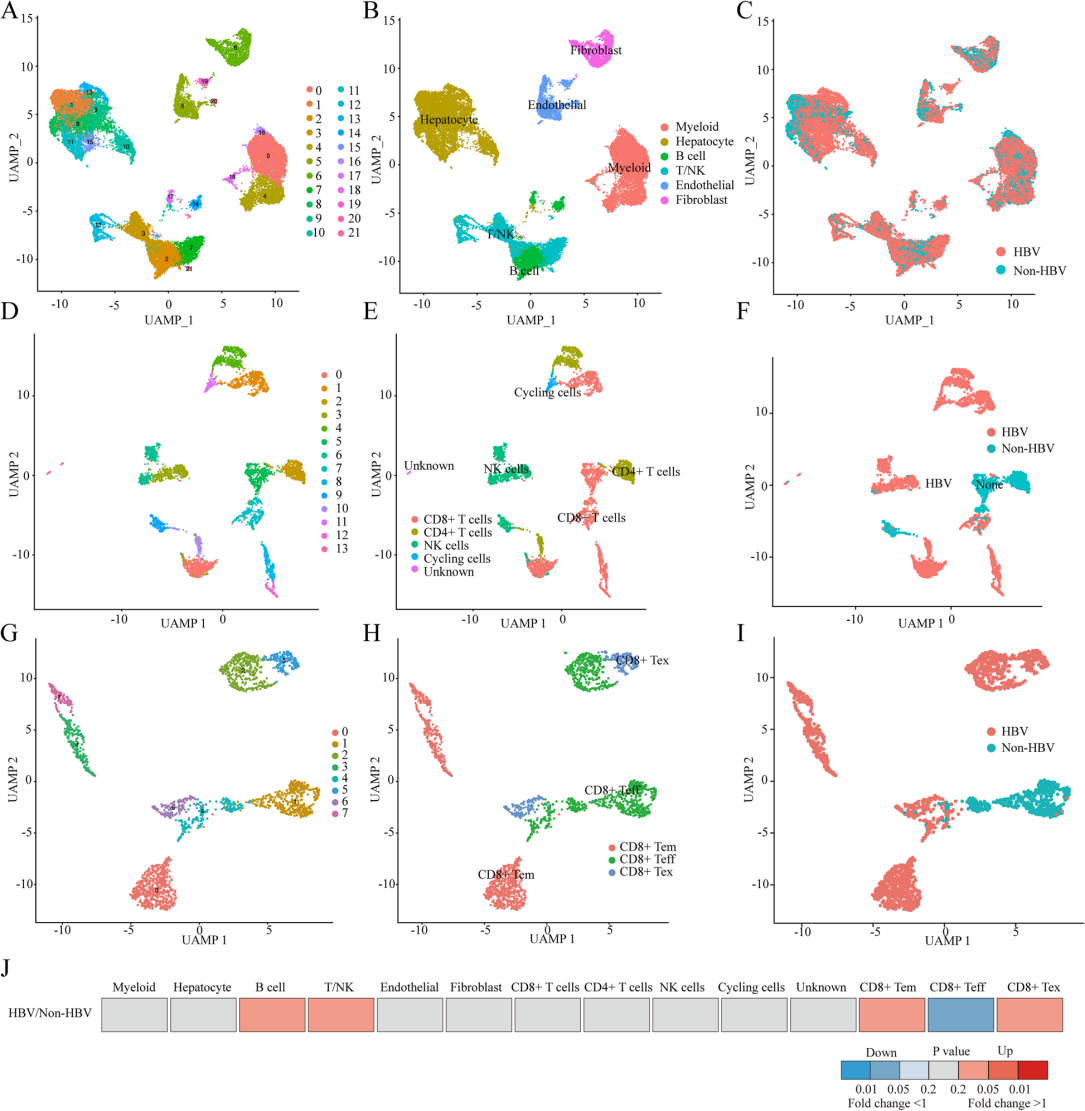
ScRNA-seq Landscape of HBV-related HCC Tumor Microenvironment. **A**, **B** UMAP plot of cells colour-coded for the indicated cell types in the microenvironment of HBV-related HCC, displaying 6 major cell types and a total of 21 subtypes. **C** UMAP plot showing cell origins by colour, the origin of HBV types. **D**, **E** UMAP plot showing the subtypes and corresponding annotations of T/NK cells in HCC patient. **F** UMAP plot of T/NK cells from the two groups of melanoma samples of HBV infection. **G**, **H** UMAP plot showing the subtypes and corresponding annotations of CD8^+^ T cells in HCC patient. **I** UMAP plot of CD8^+^ T cells from the two groups of melanoma samples of HBV infection. **J** The fold changes of the percentages of each of the  cell types comparing the HBV positive to the negative HCC

Initially, we determined the available dimensions and screened related genes using principal component analysis (PCA). We selected 20 initial principal components, followed by UMAP (Figure S1A and B). After quality control and batch effect correction, we normalized the HCC scRNA-seq data, which included 25,479 genes and 24,859 cells from the HCC samples (Figure S1C and D). Next, we conducted a cluster classification analysis of all the cells, identifying 21 separate clusters in the HCC cell population (Fig. 2A). These clusters were then annotated by cell type based on marker gene expression according to the CellMarker database and the literature (Figure S1E, F and Figure S2). The HCC cells were classified as myeloid cells, hepatocyte cells, B cells, T/NK cells, endothelial cells, or fibroblasts (Fig. 2B, C). Given the critical role of T/NK cells in the progression and prognosis of HCC, we further analysed T/NK cells and identified 13 separate clusters (Fig. 2D), which were subsequently annotated as CD8^+^ T cells, CD4^+^ T cells, NK cells, cycling cells, and unknown cells (Fig. 2E, F). Furthermore, we performed a subtype analysis of CD8^+^ T cells by categorizing them into effector, memory, and exhausted CD8^+^ T cells (Fig. 2G-I). Differential analysis of cell proportions revealed a significant decrease in the proportion of CD8^+^ Teff cells in HBV-positive HCC (Fig. 2J and Table S2). These results are consistent with previous literature suggesting that HBV infection might promote exhaustion of CD8^+^ T cells in HCC. Therefore, our subsequent studies will focus on examining the mechanisms of CD8^+^ T-cell exhaustion.

### Identification of genes associated with CD8^+^ T-cell exhaustion

**Fig. 3:**
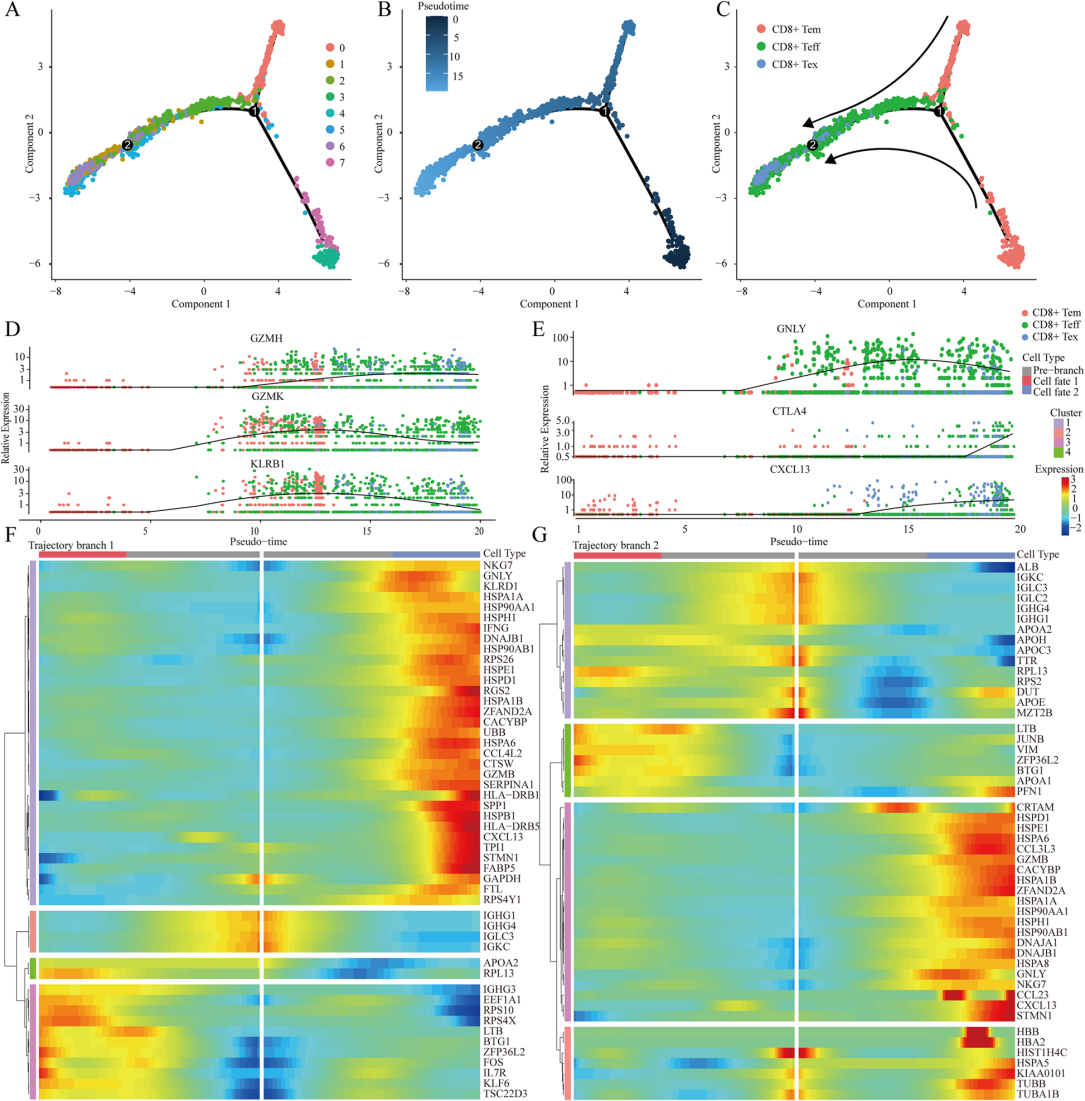
Simulation of the development trajectory of CD8^+^T cells and the analysis of gene expression pattern. **A** cell cluster transition. **B** pseudo-trajectory of CD8^+^ T cells. **C** cell type transition. **D**, **E** Expression patterns of some marker genes related to CD8^+^ T exhaustion under three types. **F**, **G** Heatmap shows the gene expression dynamics of CD8^+^ T cell types. Genes (rows) are clustered and cells (columns) are ordered according to the pseudotime development

Given the crucial role of CD8^+^ T-cell exhaustion in the development and progression of HBV-related HCC, we conducted a pseudotime analysis to simulate the trajectory of CD8^+^ T-cell exhaustion (Fig. 3A-C). Individual cells were sorted according to their marker genes, and the tree-like structure of the entire lineage differentiation trajectory was constructed using the R package ‘monocle’ (Fig. 3B). CD8^+^ Tem cells were found to be located at the initiation of trajectory branch 1, while Teff cells emerged towards the end. Teff cells appeared at the commencement of trajectory branch 2, whereas Tex cells were observed towards this conclusion (Fig. 3C). Furthermore, changes in the expression of marker genes in different states of CD8^+^ T cells were analysed. At the initial stage, the expression of the marker genes was upregulated in CD8^+^ Tem cells (GZMK and KLRB1) (Fig. 3D). Later, the expression levels of CD8^+^ Tem cell marker genes decreased, whereas the expression of marker genes of CD8^+^ Teff cells (GZMH and GNLY) significantly increased (Fig. 3D, E). Eventually, CTLA4 and CXCL13, both of which are marker genes for CD8^+^ Tex cells, were significantly upregulated at the terminal stage (Fig. 3E). These results demonstrated how CD8^+^ T cells transform from memory cells to effector cells and eventually become exhausted. Additionally, clustering of the top 50 genes according to pseudotemporal expression patterns was performed. According to the heatmap of trajectory branch 1, genes in Cluster 1 were significantly enriched in the process of converting memory cells to effector cells in cell fate 2 (Fig. 3F). Moreover, genes in Clusters 3 and 4 were significantly enriched in the process of converting effector cells to memory cells in cell fate 2, as observed in the heatmap of trajectory branch 2 (Fig. 3G). These results demonstrated the genetic characteristics of CD8^+^ T-cell exhaustion, and a total of 3,172 total T-cell exhaustion-related genes (TERGs) were identified. Therefore, further analysis was performed to examine the clinical value of genes whose expression changed during the conversion of CD8^+^ Tem cells to Teff cells and of those that changed from CD8^+^ Teff cells to Tex cells in the two trajectories.

### The TEI was established based on five TERGs

**Fig. 4:**
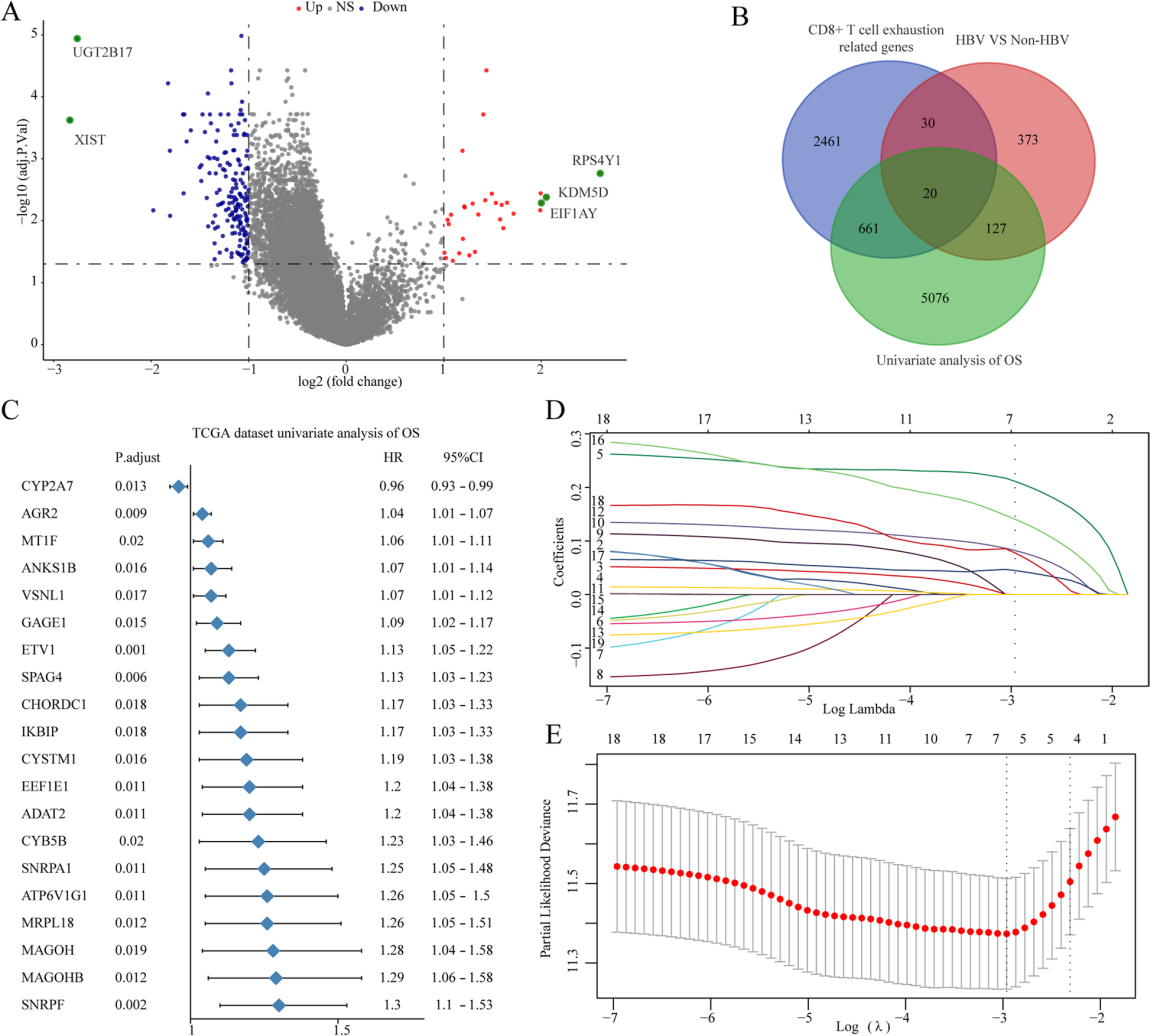
Construction of the TEI of CD8^+^T cell exhaustion-related genes (TERGs). **A** The volcano diagram depicting the differential expression of genes between HBV-negative and HBV-positive HCC samples. **B** 20 TERGs that exhibit differential expression between HBV-positive and HBV-negative HCC patients, and have the potential to impact the prognosis of HCC. **C** Univariate Cox analysis of OS in HCC. **D** Selection of the optimal parameter (lambda) in the LASSO model. **E** LASSO coefficients of the 20 TERGs in TCGA cohort

**Fig. 5:**
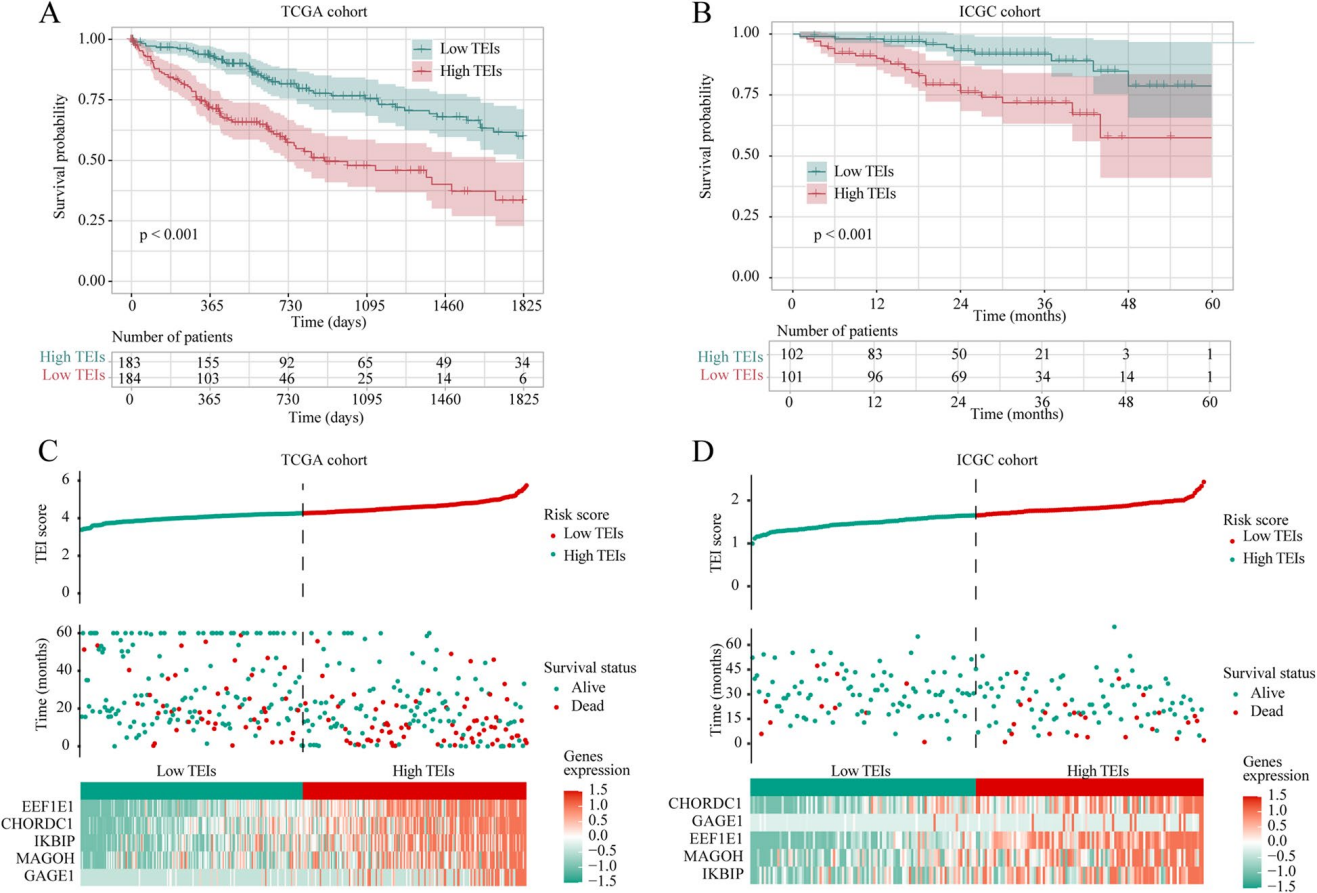
Internal training and external validation of the TEI. **A**, **B** Overall survival in the low- and high-TEI group patients in TCGA and ICGC cohorts. **C**, **D** Overall survival analysis for high-risk and low-risk groups in the training (TCGA) cohort and validation (ICGC) cohort, respectively

Considering the significant role of CD8-positive T-cell exhaustion in the prognosis and immunotherapy efficacy of HBV-related HCC, we screened prognosis-related TERGs in HCC and constructed a prognostic model. A volcano plot of the TCGA cohort showed that 183 genes were significantly downregulated and 32 genes were significantly upregulated in HBV-related HCC tissues (Fig. 4A). Subsequently, we proceeded with further analysis by selecting 20 TERGs that exhibited differential expression between HBV-positive and -negative HCC patients and could impact the prognosis of HCC patients (Fig. 4B). Univariate Cox analysis revealed that CYP2A7 may play a protective role, while the remaining genes were identified as potential risk factors for the prognosis of HCC (Fig. 4C). To accurately evaluate the prognostic value of CD8^+^ T-cell exhaustion in individual HCC patients, the CD8^+^ T-cell exhaustion indices (TEIs), which included EEF1E1, GAGE1, CHORDC1, IKBIP and MAGOH, were calculated via the LASSO Cox regression model based on the minimum criterion (Fig. 4D, E). We calculated the TEIs using the following equation: TEIs = (0.211 × expression of EEF1E1) + (0.082 × expression of GAGE1) + (0.141 × expression of CHORDC1) + (0.045 × expression of IKBIP) + (0.078 × expression of MAGOH) in the TCGA (training) and the ICGC (validation) cohorts. To further explore the prognostic value of TEIs, HCC patients were divided into high- and low-TEI groups based on the median values in the TCGA and the International Cancer Genome Consortium (ICGC) cohorts. Survival analysis of patients in the TCGA and ICGC cohorts demonstrated that high TEAD gene expression was closely associated with poor OS (Fig. 5A, B). Additionally, analyses showed that, compared with those in the high-TEI subgroup, the low-TEI subgroup had a lower number of deaths (Fig. 5C, D). In summary, TEIs can potentially predict patient prognosis.

### Correlation of TEIs with clinical characteristics and its predictive value in immunotherapy

**Fig. 6:**
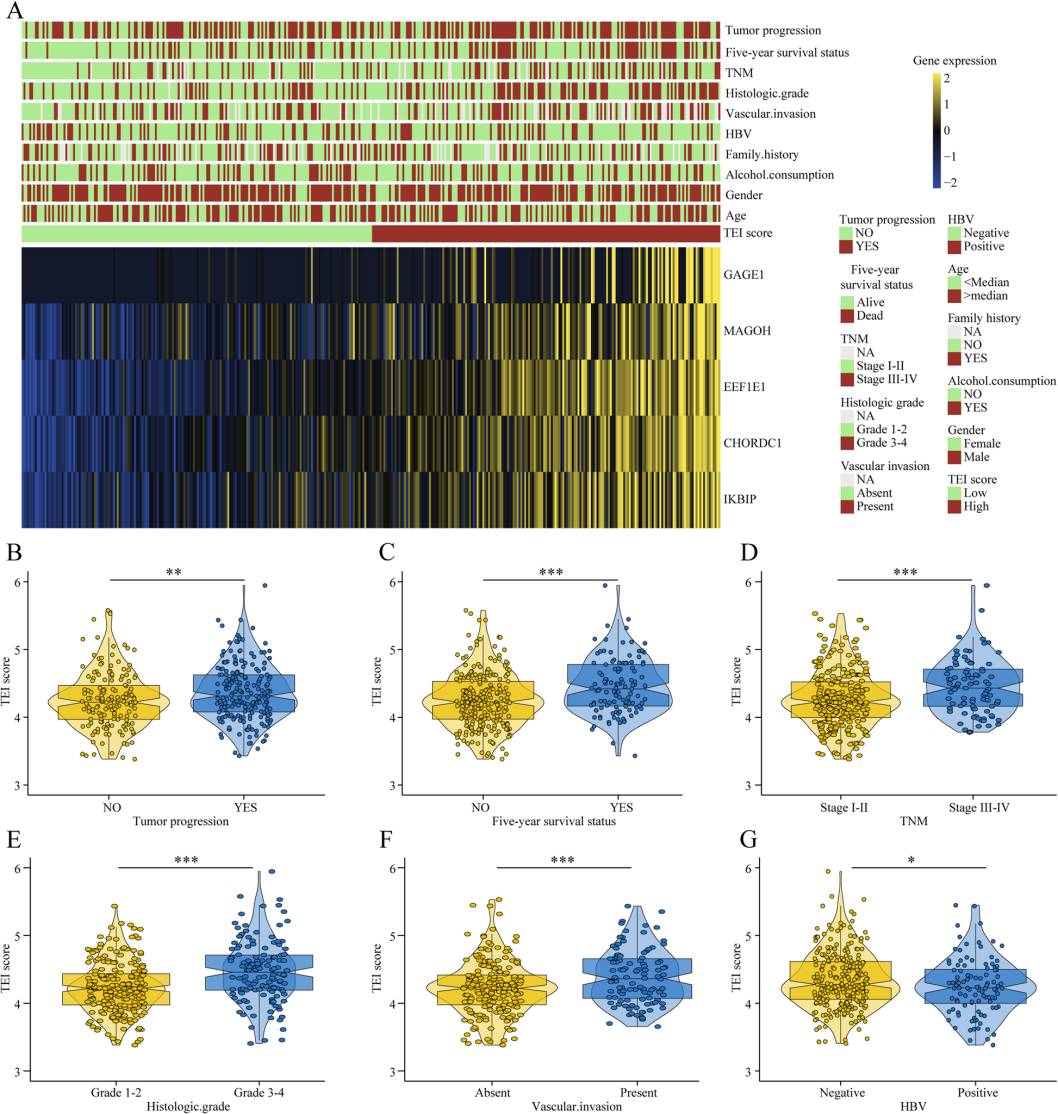
The relationship between the TEI and clinical characteristics in HCC patients. **A** Heat map of five TERGs expression and corresponding clinicopathological features of low- and high-TEI group. **B** The relationships between the TEI and clinical characteristics including tumor recurrence, Five-year survival status, TNM, histologic grade, vascular invasion and HBV infection

**Fig. 7:**
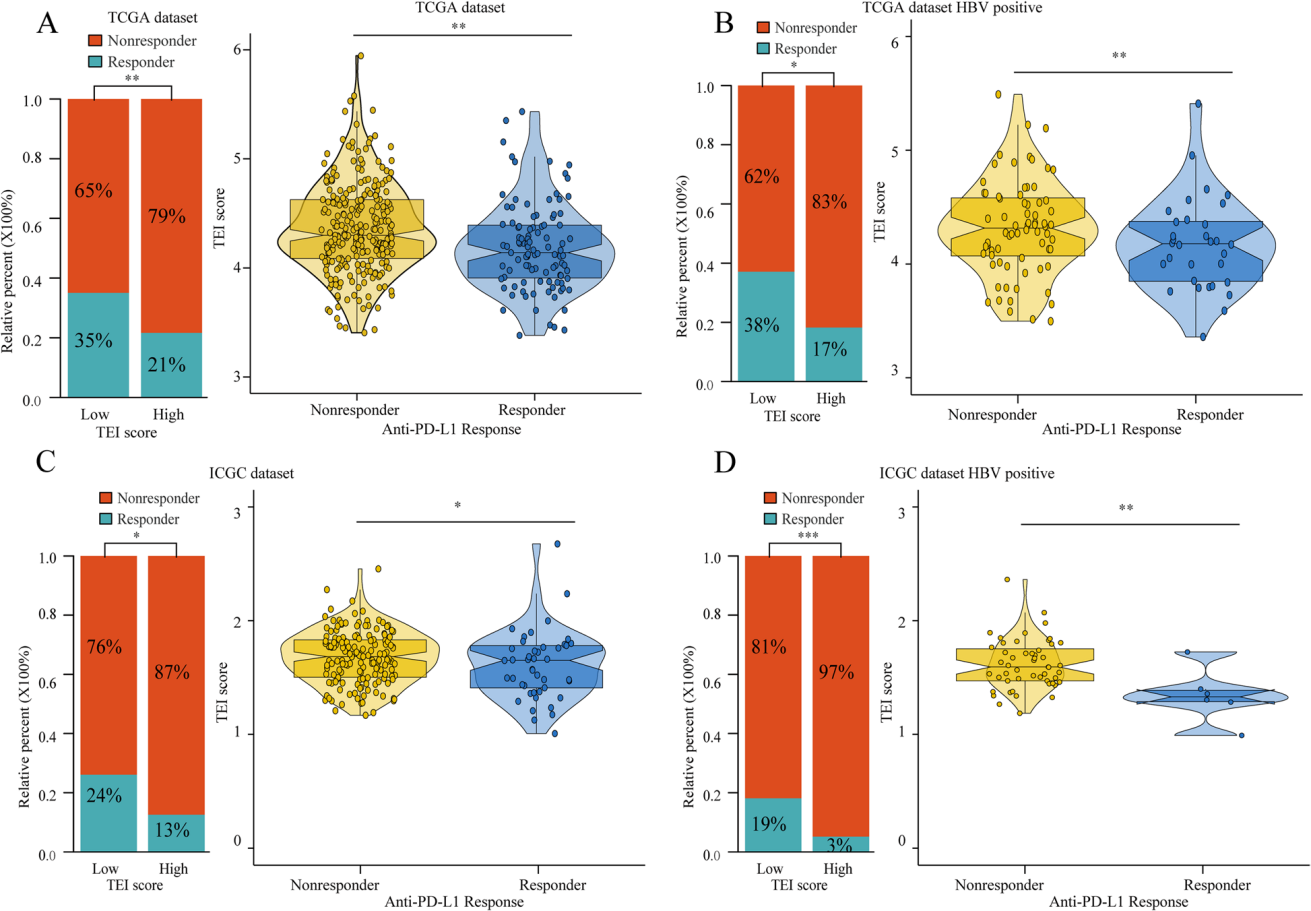
The immunotherapy value of TEI. The proportion of patients with response to immunotherapy in low or high TEI groups and the differential analysis of TEI between immunotherapy responders and non-responders were evaluated in HCC (**A**, **C**) and HBV-positive HCC (**B**, **D**) using both TCGA and ICGC datasets

We subsequently tested the correlation between clinical characteristics and TEIs. The expression of genes and clinical characteristics of patients with low- and high-TEIs are represented via heatmaps (Fig. 6A). We found that high AFP levels, high vascular invasion, high histologic grade (G3-G4), advanced TNM stage and poor five-year prognosis were closely associated with high-TEI HCC patients (Table S3). Subsequently, we evaluated the differences between the TEIs and each clinical feature. We found that high TEAD1 expression was closely associated with tumor progression, 5-year survival, TNM stage III-IV, pathological T3-4 stage, vascular invasion and HBV infection (Fig. 6B-G). Furthermore, we analysed the relationship between TEIs and immunotherapy response. We used the TIDE algorithm to predict the likelihood of response to immunotherapy based on the TCGA and ICGC cohorts. We were very delighted to see that patients with low TEIs had a more promising response to immunotherapy among all HCC patients and HBV-positive patients. Moreover, patients who responded to immunotherapy had lower TEIs (Fig. 7A-D). Overall, our study indicates that TEIs might be potential biomarkers for evaluating immunotherapy efficacy and clinical progress in patients with HBV-related HCC.

### Establishment and assessment of TEIs-correlated clinicopathologic nomogram

**Fig. 8:**
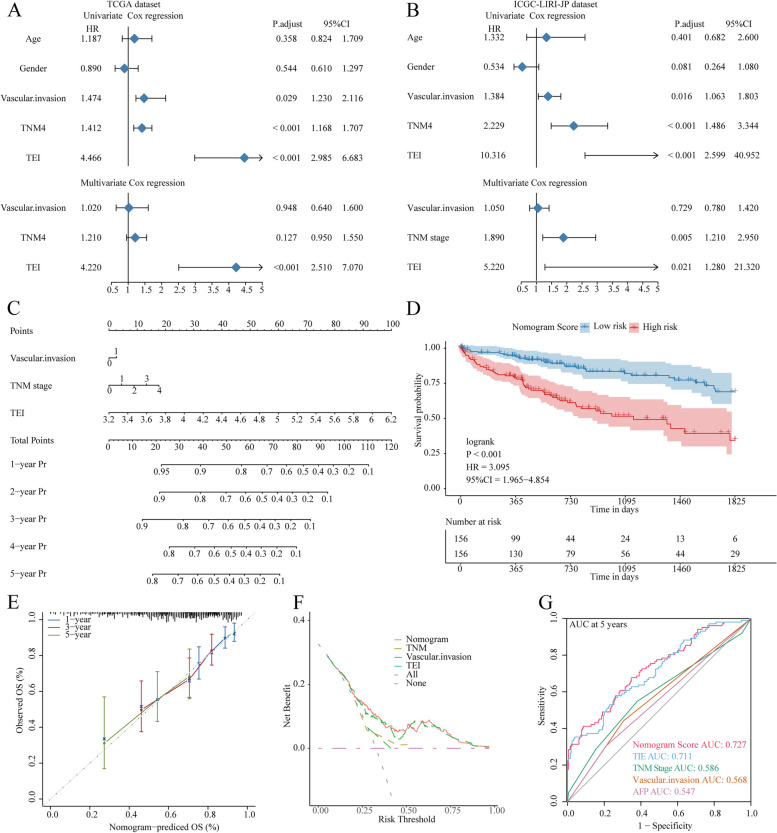
Establishment and assessment of the nomogram survival model. **A**, **B** Univariate and multivariate Cox analysis for the clinicopathologic characteristics and TEI in TCGA and ICGC cohort. **C** A nomogram was established to predict the prognostic of HCC patients. **D** Kaplan-Meier analyses for the two HCC groups based on the nomogram score. **E** Calibration plots showing the probability of 1-, 3-, and 5-year overall survival in TCGA cohort. **F** Decision curve analysis (DCA) of nomogram predicting 5-year overall survival. **G** Receiver operator characteristic (ROC) analysis of nomogram predicting 5-year overall survival in TCGA

To elucidate whether the TEI was an independent prognostic indicator of HCC, univariate and multivariate Cox regression analyses were conducted, and the results showed that vascular invasion, TNM stage and TEI were closely linked to the OS of HCC patients according to univariate Cox analysis. Multivariate analysis also indicated that TEIs were still an independent prognostic factor in HCC patients (Fig. 8A, B). A nomogram model was constructed with the TCGA cohort utilizing multivariate Cox and stepwise regression analyses to evaluate 1-, 2-, 3-, 4-, and 5-year overall survival (OS). The model incorporated vascular invasion, TNM stage, and the TEI as significant variables (Fig. 8C). A substantial difference in survival between the high-grade and low-grade groups was observed based on the nomogram score (Fig. 8D). The accuracy of this model in estimating the 1-, 3-, and 5-year survival rates was confirmed by calibration curves (Fig. 8E). Decision curve analysis (DCA) revealed that the nomogram model was superior to any other predictor utilized in our study (Fig. 8F). Furthermore, the area under the curve (AUC) values for the TCGA and ICGC cohorts were evaluated, and the results indicated that the nomogram has remarkable precision in predicting 5-year survival in HCC patients (Fig. 8G). In conclusion, based on the above findings, our prognostic nomogram for OS prediction is reliable and suitable for application in HCC patients.

### Mechanism analysis of HBV-induced exhaustion of CD8^+^ T cells

**Fig. 9:**
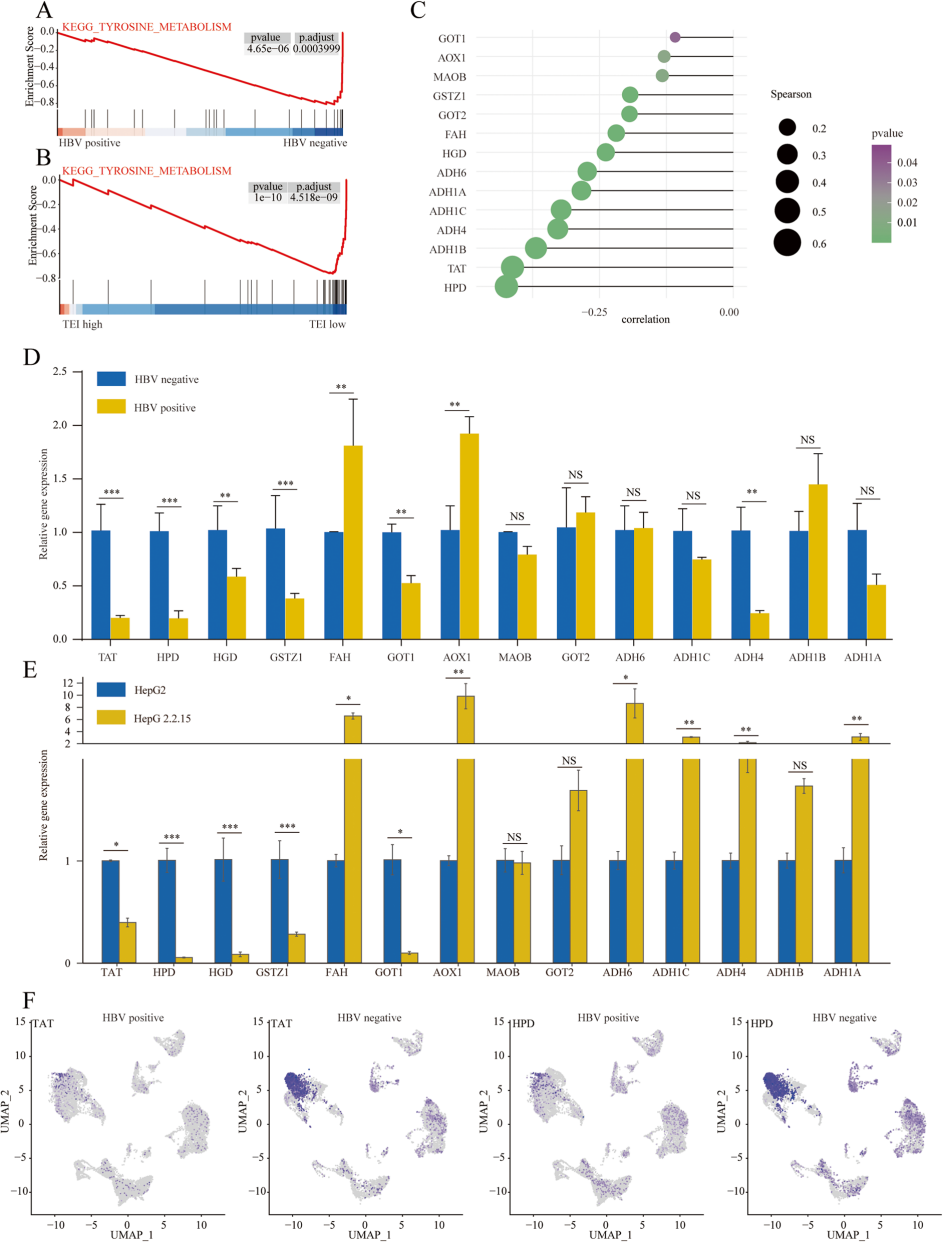
Mechanism analysis of HBV-induced exhaustion of CD8^+^T lymphocytes. **A**, **B** KEGG enrichment pathways analysis of tyrosine metabolism in TEI high and HBV-positive HCC patients. **C** Correlation analysis of TEI and tyrosine metabolism related molecules. **D** PCR experiments of tyrosine catabolism related enzymes in HBV-positive and HBV-negative HCC tissues. **E** PCR experiments of tyrosine catabolism related enzymes in HepG2 and HepG2.2.15. **F** UMAP plot displays differential expression of TAT and HPD in HBV-negative and positive HCC

To investigate the potential mechanisms by which HBV promotes immune suppression, we performed pathway enrichment analysis on different cell types. First, we extracted marker genes of different cell types from HBV-positive and -negative HCC samples and performed enrichment analysis. The results were as follows: in B cells, the “Th17 cell differentiation” pathway was significantly enriched in HBV-negative samples; in myeloid cells, the “human immunodeficiency virus 1 infection” pathway was significantly enriched in HBV-positive samples; and in T/NK cells, the “haematopoietic cell lineage” pathway was significantly enriched in HBV-negative cells. In fibroblasts, the “human cytomegalovirus infection” pathway was significantly enriched in HBV-positive HCC samples, while the “complement and coagulation cascades” and “prion disease” pathways were significantly enriched in HBV-negative samples. In hepatocytes, the “tyrosine metabolism” pathway was significantly enriched in HBV-negative samples. Previous literature suggests that the accumulation of tyrosine in the tumor microenvironment can suppress the antitumor immune response. Therefore, we further explored the underlying mechanisms involved (Figure S3). GSEA confirmed that the tyrosine metabolism pathway was significantly suppressed in HBV-positive HCC samples (Fig. 9A). The bulk data showed that tyrosine metabolism was also reduced when the TEI score was high (Fig. 9B). According to our correlation analysis, TEIs were found to be significantly negatively correlated with the key enzymes involved in tyrosine catabolism (Fig. 9C). Further qRT‒PCR experiments demonstrated that the mRNA levels of key enzymes involved in tyrosine catabolism were significantly reduced in HBV-positive HCC samples and cell line (Fig. 9D and E). Single-cell data analysis showed that TAT and HPD were most significantly decreased in HBV-positive HCC samples (Fig. 9F). Therefore, HBV may promote CD8^+^ T-cell exhaustion by inhibiting the catabolism of tyrosine and promoting its accumulation.

## Discussion

Over the past two decades, clinical trials investigating HCC therapies have demonstrated that immune checkpoint inhibitor (ICI) therapy is more effective in patients with HBV-positive HCC than in those with HBV-negative HCC [24, 25]. These findings underscore the importance of obtaining a deeper understanding of the TME in HBV-related HCC. Single-cell sequencing has provided us with opportunities to gain deeper insights into the immune microenvironment of HCC. Recent studies on the immune microenvironment of HCC have shown that this tumor highly immunosuppressive and enriched with CD8^+^ Tex cells [16]. Notably, HBV-positive HCC displayed increased infiltration of CD8^+^ T cells, albeit with heightened CD8^+^ Tex cell numbers [18]. Previous research has confirmed that the extent of exhaustion of CD8^+^ T cells plays a pivotal role in determining the efficacy of immunotherapies and patient prognosis in HCC [26]. However, reliable and effective models for prognosis assessment and biomarker identification in HBV-related HCC are still lacking. A dependable prediction model is crucial for accurate selection of treatment options and prognosis evaluation in patients with HCC.

In this study, we observed a decrease in CD8^+^ Teff cell infiltration and an increase in Tem and Tex cell infiltration in HBV-positive HCC tissues. We then extracted TERGs related to the CD8^+^ T-cell exhaustion process using pseudotime analysis. By further integrating bulk RNA-Seq data, we identified TERGs that were differentially expressed between HBV-negative and -positive HCC and significantly affected HCC prognosis. Using LASSO regression, we constructed a TEI and divided HCC patients into high- and low-TEI groups based on the median value. The TEI demonstrated good predictive ability for the prognosis of HCC patients. Subsequently, univariate and multivariate Cox regression analyses revealed that the TEI was an independent prognostic risk factor in HCC patients. Additionally, the high-TEI group was associated with advanced clinicopathological characteristics and immunotherapy nonresponse. Finally, we constructed a nomogram incorporating clinical characteristics and the TEI, which performed well. Overall, our study developed a clinically applicable model that facilitates the selection of treatment options and prognostic assessment for patients with HBV-related HCC.

As a major component of the tumor microenvironment, metabolic reprogramming of tumor cells plays a crucial role in tumor progression and the response to immune therapy [27–29]. Therefore, in our mechanistic exploration, we focused on investigating the impact of changes in intrinsic tumor cell metabolism on the response to immunotherapy in different subgroups. Subsequent mechanistic explorations revealed significant enrichment of tyrosine metabolism in patients with HBV-negative HCC and patients with lower TEI scores. Further correlational analysis revealed a significant negative correlation between the TEI and tyrosine catabolic enzyme expression. Tyrosine catabolism primarily occurs in the liver, and to a lesser extent, in the kidney. In this process, tyrosine is metabolized to generate intermediates or precursors for the tricarboxylic acid (TCA) cycle and ketogenesis [30]. Subsequently, tyrosine undergoes several enzymatic reactions to be catabolized to fumarate and acetoacetate (AcAc) [31], with the aid of enzymes such as tyrosine aminotransferase (TAT), 4-hydroxyphenylpyruvate dioxygenase (HPD), homogentisate 1,2-dioxygenase (HGD), maleylacetoacetate isomerase (GSTZ1), and fumarylacetoacetase (FAH). A reduction in the expression levels and loss of function of the aforementioned enzymes can result in tyrosinemia [32, 33]. Studies have indicated that during the progression of chronic liver disease, there is a gradual increase in blood tyrosine levels [34]. Furthermore, high tyrosine levels in HCC patients are closely associated with poor prognosis [35]. Metabolomic studies have demonstrated that HBV-positive HCC patients exhibit significantly higher serum tyrosine levels than HBV-negative HCC patients [36, 37]. Research on tumor immunotherapy has shown that consuming a diet low in tyrosine and phenylalanine can improve the body’s anti-tumor immune response [38]. A decrease in the serum tyrosine concentration may facilitate T-cell proliferation and activation [39]. In melanoma, reducing the serum levels of tyrosine and phenylalanine can inhibit tumor growth and metastasis. Moreover, targeting tyrosine metabolism can enhance the efficacy of PD-1 monoclonal antibody therapy for melanoma [40]. Animal models indicate that lowering the serum concentration of tyrosine enhances the sensitivity of HCC, lung cancer, and leukaemia to chemotherapy [41]. Although several studies have established that tyrosine metabolism plays a crucial role in the immune microenvironment of HCC, the specific mechanisms involved are yet to be fully understood.

In this study, we performed qRT-PCR validation on HBV-positive and negative HCC tissues. Our findings demonstrated that the expression of key enzymes involved in the catabolic metabolism of tyrosine, namely, TAT, HPD, HGD and GSTZ1, was notably reduced in HBV-positive HCC tissues. Subsequent single-cell analysis revealed significantly decreased TAT and HPD expression in hepatocytes from HBV-positive HCC patients. As a result, we propose that HBV infection may promote CD8^+^ T-cell exhaustion by inhibiting the catabolic metabolism of tyrosine. Monitoring the levels of tyrosine in the body and adopting a low phenylalanine and tyrosine diet during immunotherapy for HBV-related HCC patients may help predict the prognosis of immunotherapy and enhance its efficacy [42]. To sum up, the CD8^+^ T-cell exhaustion signature proposed in this study can be a useful prognostic predictor for HBV-related HCC patients. These findings can significantly improve the assessment of clinical outcomes in these patients.

## Conclusion

To conclude, this study proposes the TEI as a valuable prognostic predictor for patients diagnosed with HBV-positive HCC, as it significantly improves outcome assessment and predicts immunotherapy effectiveness. Additionally, our findings suggest that HBV infection may induce CD8^+^ T-cell exhaustion via the inhibition of tyrosine catabolism, offering novel insights into therapeutic strategies for HBV-HCC.

### Supplementary Information

**Additional file 1: Table S1.** The primer sequences implemented in this study.**Table S2.** Number of different cell subtypes in various samples. **Table S3.** The relationship between the TEI and clinical characteristics in HCC patients.

**Additional file 2: Figure S1.** Dimensionality reduction, batch correction and cell subtype annotation process of scRNA-seq. (A-B) Principal component analysis (PCA) dimension reduction analysis. (C-D) Harmony sample batch correction analysis. (E) Expression of the top 10 marker genes in 21 clusters. (F) Marker genes for annotating liver cancer cell types. **Figure S2. **Marker genes for cell type annotation. (A) UMAP plot displays marker genes for hepatocyte, T/NK cells, myeloid cells, B cells, endothelial cells, and fibroblasts. (B) Heatmap shows marker genes for refined annotation of T/NK cells. (C) Heatmap shows marker genes for refined annotation of CD8+ T cells. **Figure S3.** Pathway enrichment analysis of marker genes in different cell subtypes between HBV-positive and HBV-negative HCC samples.

## Data Availability

The datasets employed and/or scrutinized during the present study can be obtained from the corresponding author upon reasonable request. The data that underlie the outcomes of the current study is obtainable on the websites of The Cancer Genome Atlas (TCGA) (https://portal.gdc.cancer.gov/), Gene Expression Omnibus (GEO) (https://www.ncbi.nlm.nih.gov/geo), and International Cancer Genome Consortium (ICGC) (https://dcc.icgc.org/).

## Abbreviations

AUC: Area under curve
AcAc: Acetoacetate
FAH: Fumarylacetoacetase
GEO: Gene Expression Omnibus
GSTZ1: Maleylacetoacetate isomerase
HBV: Hepatitis B virus
HCC: Hepatocellular carcinoma
HCV: Hepatitis C virus
HPD: 4-hydroxyphenylpyruvate dioxygenase
HGD: Homogentisate 1,2-dioxygenase
ICGC: International Cancer Genome Consortium
ICI: Immune checkpoint inhibitor
LASSO: Least Absolute Shrinkage and Selection Operator
OS: Overall survival
PT: Primary tumor
scRNA-seq: Single-cell RNA-sequencing
TAT: Tyrosine aminotransferase
TCA: Tricarboxylic acid
TME: Tumor microenvironment
TEI: CD8^+^ T cell exhaustion index
TERGs: Genes related to exhaustion of CD8^+^ T cells
Tem: Memory T cells
Tex: Exhausted T cells
Teff: T effector cells
UMAP: Uniform Manifold Approximation and Projection
UMIs: Unique Molecular Identifiers
