# Supplementary material for: Identification of CD8+ T-cell exhaustion signatures for prognosis in HBV-related hepatocellular carcinoma patients by integrated analysis of single-cell and bulk RNA-sequencing
Source: BMC Cancer. 2024 Jan 10;24:53. doi: 10.1186/s12885-023-11804-3 (PMC10777580; doi:10.1186/s12885-023-11804-3)

**FigureS1 Dimensionality reduction, batch correction and cell subtype annotation process of scRNA-seq.** (A-B) Principal component analysis (PCA) dimension reduction analysis. (C-D) Harmony sample batch correction analysis. (E) Expression of the top 10 marker genes in 21 clusters. (F) Marker genes for annotating liver cancer cell types.


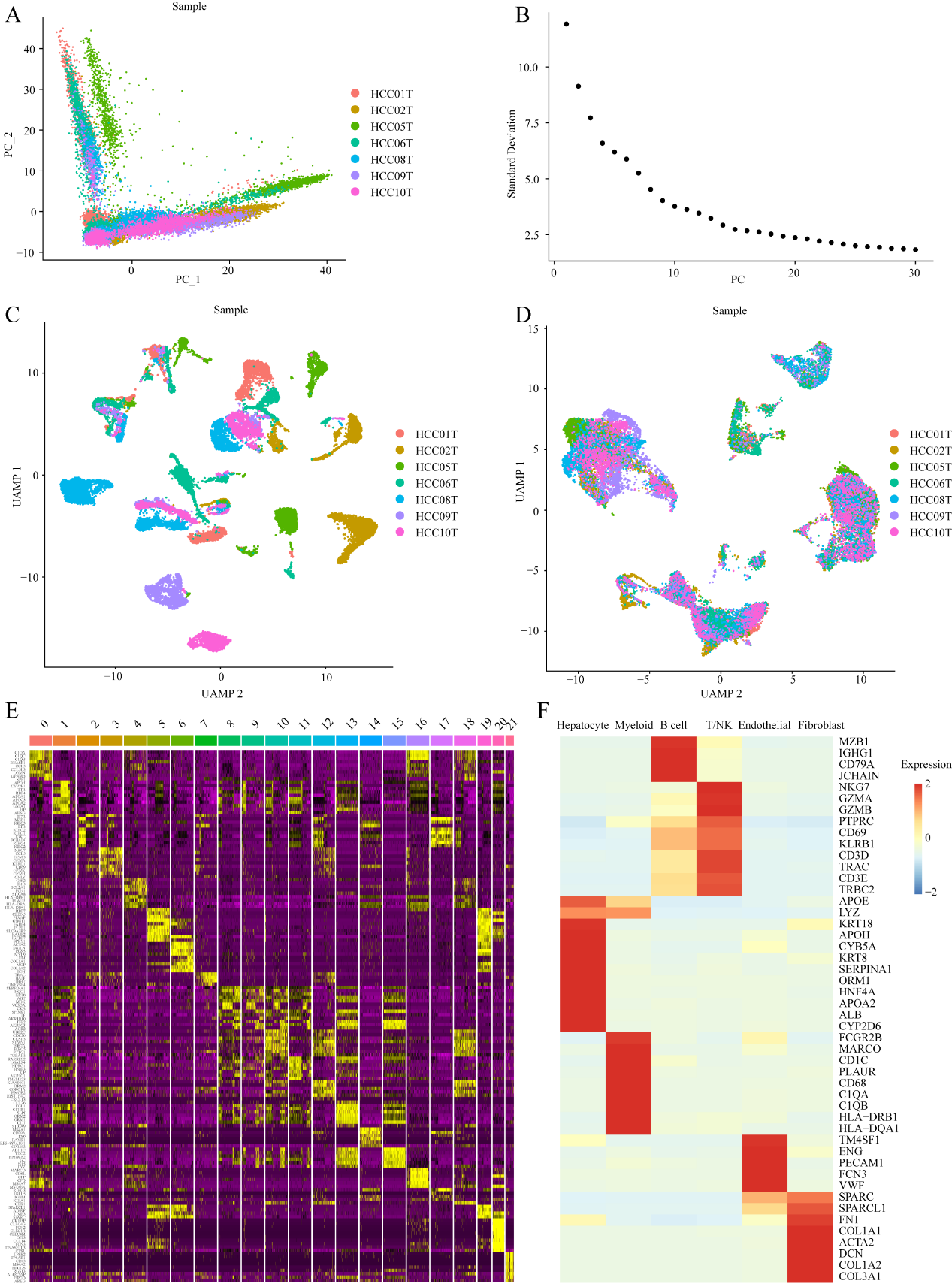


**Figure S2. Marker genes for cell type annotation.** (A) UMAP plot displays marker genes for hepatocyte, T/NK cells, myeloid cells, B cells, endothelial cells, and fibroblasts. (B) Heatmap shows marker genes for refined annotation of T/NK cells. (C) Heatmap shows marker genes for refined annotation of CD8+ T cells.


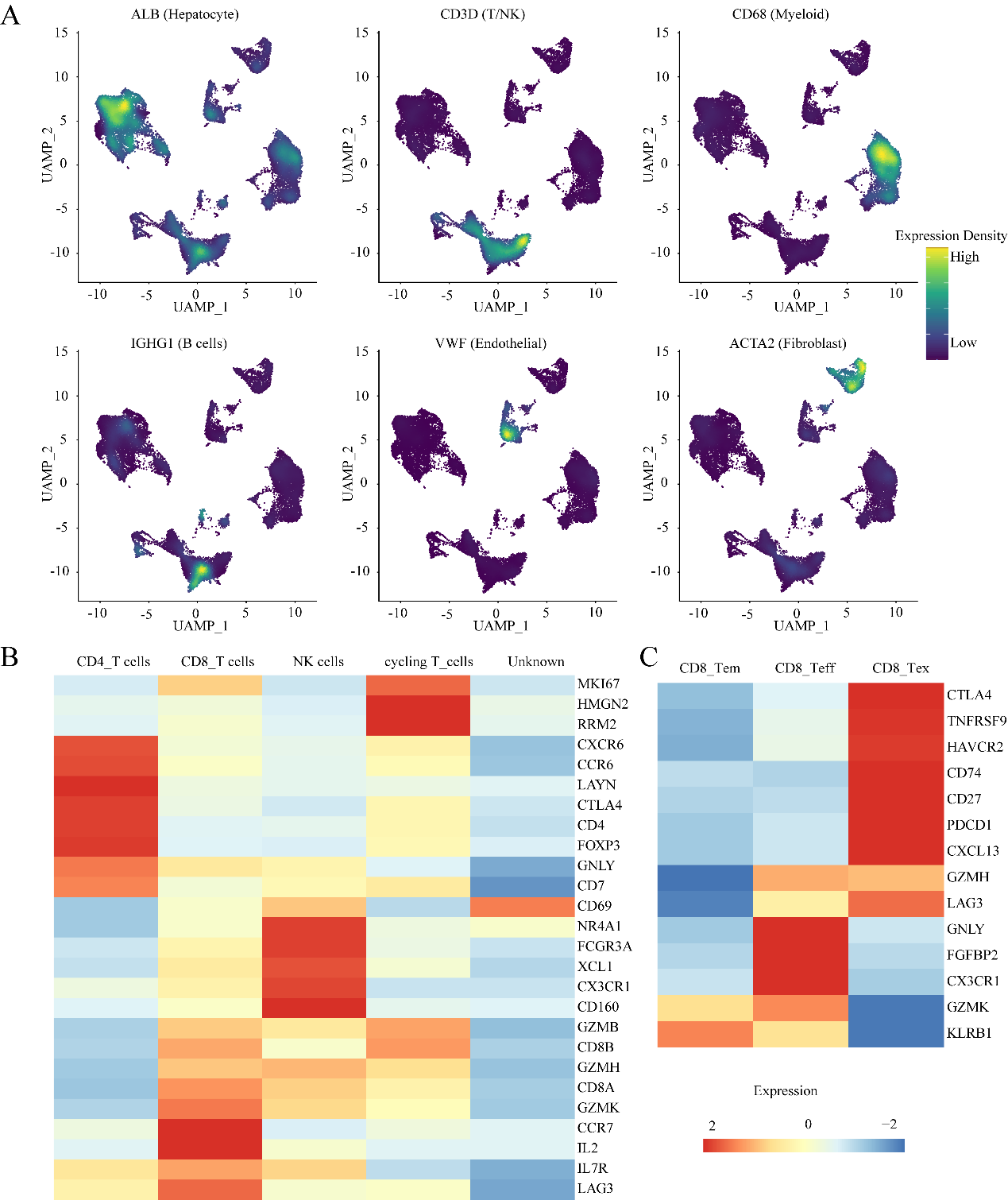


**Figure S3. Pathway enrichment analysis of marker genes in different cell subtypes between HBV-positive and HBV-negative HCC samples.**


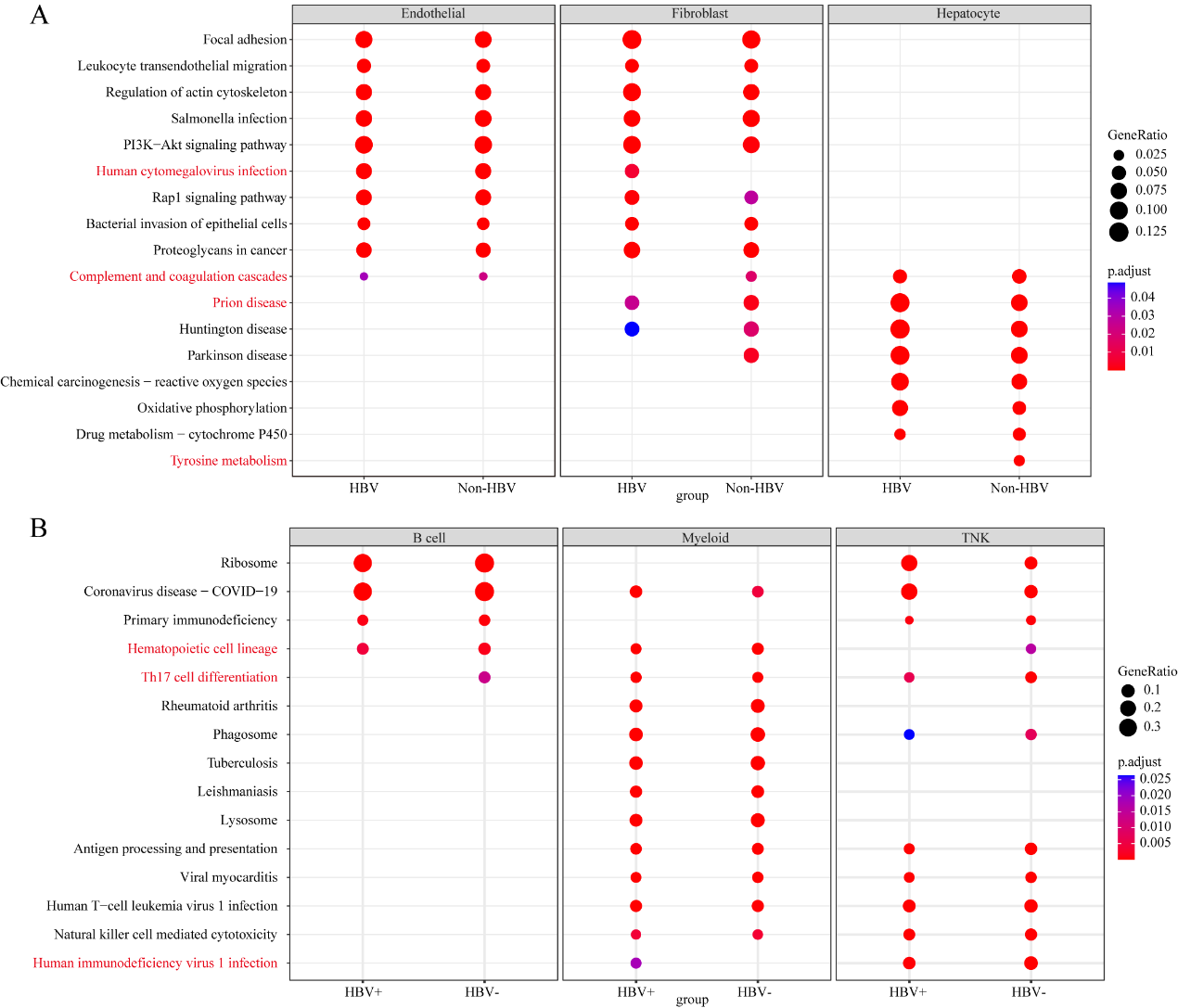

Supplement: Supplementary file 2 — Additional file 2: Figure S1. Dimensionality reduction, batch correction and cell subtype annotation process of scRNA-seq. (A-B) Principal component analysis (PCA) dimension reduction analysis. (C-D) Harmony sample batch correction analysis. (E) Expression of the top 10 marker genes in 21 clusters. (F) Marker genes for annotating liver cancer cell types. Figure S2. Marker genes for cell type annotation. (A) UMAP plot displays marker genes for hepatocyte, T/NK cells, myeloid cells, B cells, endothelial cells, and fibroblasts. (B) Heatmap shows marker genes for refined annotation of T/NK cells. (C) Heatmap shows marker genes for refined annotation of CD8+ T cells. Figure S3. Pathway enrichment analysis of marker genes in different cell subtypes between HBV-positive and HBV-negative HCC samples. [file 12885_2023_11804_MOESM2_ESM.docx]
